# Typhoid Conjugate Vaccine: A Boon for Endemic Regions

**DOI:** 10.7759/cureus.56454

**Published:** 2024-03-19

**Authors:** Nitesh Agarwal, Naveen Gupta, Nishant, Surendra H.S., Trayambak Dutta, Manish Mahajan

**Affiliations:** 1 Department of Pediatrics, Southern Gem Hospital, Hyderabad, IND; 2 Department of Pediatrics, Happy Family Hospital, Karnal, IND; 3 Department of Pediatrics, Nihan Medical Children Hospital, Patna, IND; 4 Department of Pediatrics, Natus Women and Children Hospital, Bengaluru, IND; 5 Department of Infectious Disease, Zydus Lifesciences, Ahmedabad, IND; 6 Medical Affairs, Zydus Lifesciences, Ahmedabad, IND

**Keywords:** tetanus toxoid (tt), salmonella paratyphi, salmonella typhii, typhoid conjugate vaccine, crm197, vips, endemic regions, vaccine, typhoid, tcv

## Abstract

Typhoid fever has the highest disease burden in countries in low- and middle-income countries, primarily located in Asia and Sub-Saharan Africa. Previous typhoid vaccines such as the live attenuated typhoid (Ty21a) vaccine and Vi (virulence) capsular polysaccharide vaccine had the limitation that they could not be administered with other standard childhood immunizations and were ineffective in children under two years of age. To address these shortcomings of the previous vaccines, typhoid conjugate vaccines (TCVs) were developed and prequalified by the World Health Organization. Cross-reacting material and tetanus toxoid are widely used as carrier proteins in TCVs. According to various studies, TCV has higher efficacy, has a more extended protection period, and is safe and immunogenic in infants as young as six months. This review article aims to comprehensively appraise the data available on TCVs’ efficacy, duration of protection, safety, and immunogenicity in endemic regions.

## Introduction and background

*Salmonella typhi *is the leading pathogen for bacterial bloodstream infections in children under two months of age, accounting for nearly two-thirds of all bacterial isolates in many endemic countries [1]. Hence, in endemic countries, due to resource-constrained settings, typhoid represents an extensive burden on the overall healthcare system [2]. Low- and middle-income countries (LMICs), particularly in Asia and sub-Saharan Africa, bear most of the global burden of typhoid [3,4]. Each year, 11.9 to 26.9 million cases of typhoid fever are reported in LMICs [5]. An updated modeling study of 2019 reported 9.2 million typhoid fever cases and 110,000 deaths worldwide, where the highest estimated incidence was in the Southeast Asian (306 cases per 100,000 persons) regions [6]. According to the World Health Organization (WHO), countries with endemic typhoid fever should establish health facility-based surveillance and laboratory confirmation to detect outbreaks quickly, monitor antimicrobial resistance patterns, and assess the effect of vaccination [7]. Vaccination against typhoid proved effective when combined with other preventive measures, such as hand-washing, treating household water, and ensuring adequate sanitation [8]. Induction of the vaccine should be accompanied by health education, improved water, sanitation, and hygiene (WASH), and appropriate training of healthcare workers in diagnosing and treating typhoid [9]. In October 2017, the WHO strategic advisory group of experts issued new recommendations in support of the usage of TCV in endemic countries with a high disease burden or antimicrobial-resistant *S. typhi* [10].

The outbreak of typhoid fever caused by *Salmonella enterica serovar Typhi* (*S. typhi*), a gram-negative bacterium, is one of the most serious public health concerns. The most common agents of enteric fever are *S. typhi *and *Salmonella serovar paratyphi A* [11]. After invading the small intestine, S. typhi colonizes macrophages of the reticuloendothelial system, which is followed by their shedding into the bloodstream [12,13]. Symptoms of typhoid include fever, malaise, anorexia, abdominal pain, and other gastrointestinal symptoms [14]. Untreated or improperly treated infections can result in septic shock, gastrointestinal hemorrhage, or intestinal perforation, resulting in 1%-4% mortality with treatment and 10%-20% without treatment [15]. The range of mortality is estimated to be between 1% and 5% in *S. typhi* infected hospitalized patients, whereas symptoms were found to subside typically in 7-21 days [16]. A chronic carrier state can also develop when the bacteria colonize the gallbladder [17]. *Salmonella typhi *is a human host-restricted organism [11]. The Global Alliance for Vaccines and Immunization (Gavi) pledged $85 million to assist with the rollout of TCVs in Gavi-eligible countries [18]. Therefore, the current review article focuses on a comprehensive appraisal of the data available on efficacy, duration of protection, safety, immunogenicity, and other clinical outcomes of TCV in endemic regions.

## Review

Typhoid vaccines

The following three typhoid vaccines are currently available: live attenuated typhoid (Ty21a) vaccine, Vi (virulence) capsular polysaccharide (Vi-PS) vaccine, and typhoid conjugate vaccine (TCV).

Live Attenuated Ty21a Vaccine

Ty21a is the first live oral attenuated *Salmonella *vaccine in the form of liquid and enteric-coated capsule (e.g., Vivotif® by Crucell and PaxVax) developed by chemical mutagenesis of wild-type Ty2 strain of *S. typhi* in Switzerland [19]. To address the limitations associated with killed whole-cell vaccine, development of more competent vaccine candidate was necessary. Live attenuated strains, which lacked the galactose-epimerase gene and the Vi antigen, were considered for their greater immune response [8]. For Ty21a vaccine, several trials have confirmed the efficacy of 67% for more than seven years [20]. Despite a satisfactory immune response and efficacy, Ty21a had several limitations such as 1) a high number (109) of bacteria are required for the oral dose to achieve adequate immunity; 2) the vaccine is recommended for children aged more than five to six years of age, and 3) Ty21a is highly acid-labile, thus stomach acidity is needed to be neutralized or bypassed on oral administration. It should be mentioned here that Ty21a is not a WHO-prequalified vaccine [8,20,21].

Vi Capsular Polysaccharide (Vi-PS) Vaccine

The Vi-PS vaccine is an injectable subunit vaccine, derived from the Ty2 *S. typhi *strain, which was first licensed in 1994 [19]. The immunogenicity of the vaccine is 65% (moderate), and repeat dosing is required every three years [22]. Typhim Vi is an example of Vi-PS vaccine, which was manufactured by Sanofi Pasteur (Lyon, France). Typhim Vi vaccine was prequalified by WHO in 2011. Other examples of Vi-PS vaccine include Typherix® (manufactured by GlaxoSmithKline [GSK], Rixensart, Belgium]) and Typbar® (manufactured by Bharat Biotech, Hyderabad, India) [20]. The injectable Vi-PS are intramuscularly administered as a single dose. Protection is induced about seven days after the injection. In endemic regions, the protective efficacy is 72% after 1.5 years of vaccination, and after three years, it is about 50% [23]. Limitations of the Vi-PS vaccines include failure to induce T-cell-independent immune response, poor immunogenicity in young children (especially for those aged less than two years) [7], and need for repeat dosing after an interval; Vi-PS vaccine is administered parenterally as a single dose and is followed by high levels of seroconversion of serum IgG anti-Vi antibodies [24].

Need for the development of TCV

The challenges associated with Ty21a and Vi polysaccharide vaccines are evident from the following facts. Firstly, both are ineffective in children below two years of age; nevertheless, there is conflicting evidence regarding the efficacies of these vaccines in the group of children aged two to five years [25]. Studies on the effectiveness Vi-PS typhoid vaccine were carried out in the low socio-economic parts of the cities of Kolkata (India) and Karachi (Pakistan) [26,27]. In Kolkata, the vaccine was found highly effective in the children in the age group of two to five years with good herd protection, but the same vaccine was found to fail in providing any protection and herd effect in the younger age group (2 to 5 years) in Karachi [27]. Secondly, re-vaccination is needed for both the vaccines. Being purely polysaccharide vaccines, they failed to induce T-cell mediated immunity; consequently, there was no immune memory, and extra doses were required for repeated re-vaccinations [28]. Thirdly, the polysaccharide vaccine mediated antibody response was found to result in low titers of poor affinity IgG antibodies [25]. Fourthly, there is a possibility of hypo-responsiveness with succeeding doses of ViPS vaccines [28]. Therefore, co-administration of the Vi-PS vaccine with other standard childhood vaccines provided under an expanded program on immunization is not recommended [25].

To overcome the limitations mentioned above of vaccines, WHO has prequalified TCV. These vaccines can be administered to children aged six months and above and incorporated into routine immunization programs [24]. TCV single dose appeared safe and 79-95% effective, and the antibody response continued for up to seven years when administered to children [9]. Co-administration of TCV does not interfere with the immune response of routinely administered vaccines. TCV vaccination is cost-effective for countries with a high incidence of typhoid fever [9,24].

Presently, as per the WHO, programmatic utilization of TCV is recommended for typhoid fever control and prioritization of vaccine initiation in the countries with the highest incidence of typhoid fever or in countries where a higher prevalence of antimicrobial-resistant *S. typhi* is seen [9]. Typhoid vaccination rates in endemic areas have historically been low due to the modest efficacy, short period of protection, and inability to administer to young children [29]. However, the new TCV is proven to provide high and prolonged immunogenicity, and it can be administered to infants as young as six months, thereby allowing for their inclusion into the national immunization programs. These vaccines use the carrier protein-conjugated Vi-PS [4,30,31].

Interestingly, researchers have also found that *S. typhi* expresses a regulated virulence with the capsular polysaccharide on its surface, leading to the development of TCV. The research began in the 1980s and involved assessing multiple carrier proteins. Vi polysaccharide or Vi-PS, purified from the surface, was immunogenic and offered reasonable protection for adults and children over three years old [24,31].

Typhoid conjugate vaccine

The conjugation of polysaccharides to a carrier protein overcomes the limitations of polysaccharide vaccines by transforming the immune response into T-cell dependent immune response, leading to affinity maturation, subclass switching, and induction of memory [32].

Prototype conjugate vaccine: Vi-rEPA

Vi-rEPA was developed by the conjugation of Vi polysaccharide to a non-toxic recombinant protein antigenically identical to Pseudomonas aeruginosa exotoxin A (rEPA), utilizing N-succinimidyl-3-(2-pyridyldithio)-propionate or adipic acid dihydrazide as linker [8,33]. A single intramuscular dose of Vi-rEPA provides antibodies for up to four years in children aged two to five years [25].

Typbar-TCV: Bharat Biotech

Typbar-TCV consisted of 25µg (microgram) of Vi polysaccharide from *S. typhi* conjugated to tetanus toxoid (TT) carrier protein in isotonic saline and licensed for single intramuscular dose administration for those aged ≥6 months to 45 years [20]. Typbar-TCV was developed using TT as the carrier protein with Vi polysaccharide by Bharat Biotech in Hyderabad, India, which received prequalification by WHO in January 2018 [8]. It is claimed that Typbar-TCV protects from typhoid for ≥5 years after primary immunization [25]. The vaccine was found to have an efficacy of 54.6% based on data of persistent fever and an efficacy of 87.1% based on post hoc analysis of alternative diagnostic criteria of persistent fever followed by positive blood culture [25].

PedaTyph: BioMed

PedaTyph was developed by conjugation of *S. typhi *Vi polysaccharide to TT protein. It is the first TCV licensed in India (2008). Children aged more than three months of age are eligible to receive PedaTyph. At first, a single dose of 0.5 mL of this vaccine is administered intramuscularly, followed by booster dose at the age of 2.5-3 years [8,24]. It is claimed that PedaTyph imparts protection for up to 2.5 years [25].

Zyvac TCV: Zydus Lifesciences Ltd

TT was used as the carrier protein to develop Zyvac TCV by Zydus Lifesciences Ltd, Ahmedabad, India. Several trials with 238 participants in all age groups have been conducted at seven sites across India to evaluate its non-inferiority with Typbar-TCV. Vi-TT has been provided license in India for administration to children older than six months with a single dose of 25 µg. This vaccine is now licensed in India to be administered as a single dose for children older than six months [8,20].

Vi-CRM197, GVGH

GVGH (GSK Vaccines Institute for Global Health) has utilized cross-reacting material (CRM197), non-toxic mutant of diphtheria toxin (DT), as the carrier protein to develop Vi-CRM197. The safety and immunogenicity of the Vi-CRM197 vaccine against *S. typhi *have been evaluated by various phase 1 and phase 2 studies, and it was found that the Vi-CRM197 vaccine is as safe and immunogenic as Vi-PS vaccines [34]. Table 1 illustrates the impact of TCV on the clinical outcomes of typhoid fever.

**Table 1:** TCV and its impact on clinical outcomes of typhoid fever (data of five years) CRM 197, cross-reactive-material-197; ELISA, enzyme-linked immunosorbent assay; GMT, geometric mean titer; IPV, inactivated polio vaccine; JE, Japanese encephalitis; MenA, capsular group A meningococcal conjugate; MCV-A, meningococcal serogroup A conjugate vaccine; MMR, measles, mumps, and rubella; MR, measles and rubella; MV, measles vaccine; TCV, typhoid conjugated vaccine; Vi-DT, virulence diphtheria toxoid; Vi-PS, virulence capsular polysaccharide; Vi-TT, virulence tetanus toxoid; WASH, health education, improved water, sanitation, and hygiene; YF, yellow fever

Author	Study Design and Methods	Key Findings
Hoffman et al., 2023 [35]	Prospective blood culture-based surveillance study was conducted in six hospitals in Navi Mumbai, India. 81 typhoid cases and 238 matched controls were included.	To combat typhoid illness, TCV can be provided in mass vaccination campaigns.
OK Baik et al., 2023 [36]	Clinical phase II/III study (Philippines). Participants aged six months to 45 years were administered test vaccine (EuTCV) or comparator Typbar-TCV®	EuTCV in healthy volunteers possessed comparable safety and considerable immunogenicity compared to Typbar-TCV®.
Chaudhary et al., 2023 [37]	Randomized, active-controlled, immunological non-inferiority study. Participants from eastern Nepal were randomized into four study groups (A-D) within the three age strata (6 months to <2 years, 2 to <18 years, and 18 to 45 years). Groups A to C received a single dose (25 μg) of Vi-DT test vaccine, while group D received the comparator, Typbar-TCV®, and Vi-TT vaccine (25 μg) in 1:1:1:1 ratio and evaluated at four weeks postvaccination with six months follow-up.	Vi-DT vaccine was safe, immunogenic, and immunologically non-inferior to Vi-TT.
Tadesse et al., 2022 [38]	Cluster randomized trial (Bangladesh). Children (9 months to <16 years) were randomized 1:1 by cluster to Vi-TT, TCV, or JE vaccine. Surveillance for blood culture-confirmed typhoid fever was conducted over 2 years.	TCV programs coupled with WASH practices were independently associated with significantly reducing typhoid.
Kandulna et al., 2022 [39]	Open-label, phase IV extension study, India. 112 (Cadila-TCV-57, Bharat-TCV-55) adults and pediatricians were analyzed. Eligible subjects received a single dose of Cadila-TCV and were followed up for 28 days post-booster.	Persistent immunogenicity was seen after primary vaccination in subjects receiving both TCVs and robust immune response was noted after booster vaccination.
Saluja et al., 2022 [40]	Randomized, open-label, phase III study, India. 360 participants aged 9-15 months were randomized equally into Vi-DT + MMR (180 participants) or MMR alone (180 participants) group and were evaluated for safety and immunogenicity for 28 days post-vaccination.	There was a non-interference of the MMR vaccine with Vi-DT, and Vi-DT conjugate vaccine could be considered an addition to the EPI schedule among children at risk of contracting typhoid.
Thuluva et al., 2022 [41]	Multicenter, single-blind, randomized, phase II/III study, India. Comparing the immunogenicity and safety of Biological E. Ltd’s Typhoid Vi-CRM_197_ conjugate vaccine (TyphiBEV^TM^) with Vi-TT conjugate vaccine (Typbar-TCV®) in 622 healthy subjects (311 each in both vaccine groups).	TyphiBEV^TM^ was found to be non-inferior to Typbar-TCV®.
Nampota-Nkomba et al., 2022 [42]	Phase III, double-blind, parallel design, randomized controlled trial, Malawi. 200 children (9-11 months, 1-5 years, and 6-12 years) were randomly assigned (1:1) to receive TCV or control (MCV-A vaccine intramuscularly).	TCV provided safety, tolerability, and immunogenicity for up to 730-1,035 days in Malawian children aged 9 months to 12 years.
Kumar et al., 2022 [43]	Observer-blind, active-controlled, randomized, non-inferiority, phase III trial, Nepal. Participants were randomly assigned (1:1:1:1) and stratified by age (6 months to <2 years, 2 years to <18 years, and 18 years to 45 years) into four groups (A-D). Participants in groups A-C received a single dose (25 μg; 0.5 mL) of Vi-DT test vaccine via intramuscular injection from one of three good manufacturing practice lots, and those in group D received a single dose (25 μg; 0.5 mL) of the Vi-TT vaccine.	A single dose of the Vi-DT test vaccine was found to be safe, immunogenic, and non-inferior to the Vi-TT vaccine at four weeks post-vaccination.
Vadrevu et al., 2022 [44]	Phase IV, randomized, factorial assigned, open-label study, India. Typbar-TCV® was administered concomitantly with MV or MMR vaccines in 8- or 9-month-old children.	The seroconversion rates for MMR antibodies remained unaffected by concomitant administration with TCV. Typbar-TCV® could be safely co-administered with measles and MMR vaccines in children aged ≥9 months.
Choi et al., 2021 [45]	Open-label clinical phase I study, Philippines. 75 healthy adults aged 18-45 years were randomized in a 1:1:1 ratio based on the vaccines administered: EuTCV (Test), Typbar-TCV®, and Typhim.	EuTCV was found to be well-tolerated and exhibited an acceptable safety profile. Vi-CRM197 conjugate dose of 25 μg could be considered effective with a favorable safety profile.
Vadrevu et al., 2021 [46]	Phase III, open-label study, India. Anti-Vi IgG in boosted and unboosted children 3, 5, and 7 years post-primary immunization were monitored using three different ELISAs (VaccZyme™ kit ELISA [all specimens], “Szu” ELISA [all specimens], and National Institute of Biological Standards ELISA [subset]).	Administration of a booster dose of Typbar-TCV® in children ∼5 years after their primary dose, i.e., coinciding with school entry, is advisable to extend protection.
Patel et al., 2021 [47]	Phase III, double-blind trial, Malawi. Randomly assigned children who were between 9 months and 12 years of age, in a 1:1 ratio, to receive a single dose of Vi-TCV (n=14,069) or meningococcal capsular group A conjugate (MenA) vaccine (n=14,061).	TCV resulted in a lower incidence of blood culture-confirmed typhoid fever than the MenA vaccine in children aged 9 months to 12 years.
Sirima et al., 2021 [48]	Double-blind, randomized controlled trial, Burkina Faso. Children were recruited at the 15-month vaccination visit and were assigned randomly (1:1:1) to three groups. Group 1 children received TCV plus control vaccine (IPV) and MCV-A 28 days later; group 2 received TCV and MCV-A; group 3 received MCV-A and control vaccine. A routine MR vaccine was administered to all participants.	TCV generated robust immunity without interference with the MCV-A vaccine and can be safely co-administered at 15 months with MCV-A.
Sirima et al., 2021 [49]	Double-blind, randomized controlled trial, Burkina Faso. Healthy children aged 9-11 months were randomized 1:1 to receive TCV (group 1) or control vaccine (IPV, group 2).	TCV conferred robust immunogenicity without interference with MR or YF vaccines.
Shakya et al., 2021 [50]	Randomized controlled trial, Nepal. Children aged nine months to younger than 16 years were randomly assigned in a 1:1 ratio to receive a single dose of TCV (Typbar-TCV®) or MenA vaccine.	The protective efficacy of TCV against blood culture-confirmed typhoid fever at two years was 79% (p<0.0001). The incidence of typhoid fever was 72 cases per 100,000 person-years in the TCV group and 342 cases per 100,000 person-years in the MenA group.
Qadri et al., 2021 [51]	Cluster randomized trial preceded by a safety pilot phase in Bangladesh. 1,350 residents were randomly assigned (1:1) to either Vi-TT or SA 14-14-2 JE vaccine. The study population was followed for an average of 17 months.	The vaccines were well-tolerated, and no serious vaccine-related adverse events were observed. Vi-TT protects against typhoid fever in children vaccinated between 9 months to 15 years.
Kundu et al., 2020 [52]	Randomized controlled trial, India. 240 healthy subjects (6 months to 45 years). Participants received a single dose of test TCV or comparator TCV (marketed typhoid Vi conjugate vaccine) at baseline and were followed up for six weeks post-vaccination.	The immunogenicity and safety of test TCV are comparable to the existing marketed comparator TCV.
Cartee et al., 2020 [53]	Randomized, double-blinded, dose-escalating phase 1, Philippines. 15 healthy adult subjects aged 18 to 55 years were randomized and received Typhax, Typhim Vi, or a placebo (9:3:3).	Typhax was found to be safe, well-tolerated, and immunogenic.
Capeding et al., 2020 [54]	Randomized, observer-blinded phase II study, Philippines. Participants aged 6-23 months were enrolled and randomized to Vi-DT (25 µg) or placebo (0.9% sodium chloride) and evaluated for immunogenicity and overall safety 28 days post-vaccination.	Vi-DT vaccine was immunogenic, safe, and well-tolerated in children aged 6-23 months.
Medise et al., 2020 [55]	Phase II randomized, observer-blind, superiority design, Indonesia. Vi-DT TCV was compared to Vi-PS in 200 subjects aged 2-11 years.	Vi-DT vaccine was safe and immunogenic in children aged 2-11 years old.
Medise et al., 2020 [56]	Phase II interventional, blinded, comparative, randomized study, Indonesia. 200 healthy subjects were divided into trial (Vi-DT) and control groups (Inactivated poliovirus vaccine).	The novel typhoid Vi-DT conjugate vaccine was safe and immunogenic in children 6 to <24 months.
Shakya et al., 2019 [57]	Phase III, randomized controlled trial, Nepal. Randomly assigned children between 9 months and 16 years of age, in a 1:1 ratio, to receive a TCV or a MenA vaccine as a control.	A single dose of TCV was found to be immunogenic and effective in reducing *S. typhi *bacteremia in children between nine months and 16 years of age.

Vi-DT: BioFarma

Vi-DT vaccine was developed using conjugation of the Vi-polysaccharide antigen with diphtheria toxoid. It is an investigational product. With a phase 3 trial, the seroconversion rate of Vi-DT across all age strata was found to be 99.33%, with safety profile similar to Vi-TT [37,43].

Type of typhoid conjugate vaccine carrier proteins

TCV is developed by covalent attachment of an antigenic polysaccharide to a non-toxic carrier protein that boosts the immunogenicity of polysaccharide antigens and enables the host defense against encapsulated pathogens [58]. Polysaccharides coupled with protein carriers enhanced their immunogenicity as human vaccines.

Cross-Reacting Material (CRM197)

CRM197 is widely used as a carrier protein in TCV. CRM197 protein is a non-toxic DT variant with a single mutation (glycine to glutamate substitution at position 52) that removes its toxicity. CRM197 is isolated from Corynebacterium diphtheriae C7 (β197) cultures. It is non-toxic and has more lysis sidechains available for conjugation. It retains identical immunostimulant properties as DT and is used to develop safe and effective TCV for all age groups. CRM197 is synthesized as a single chain holoprotein, comprising fragment A (catalytic domain) and fragment B (transmembrane domain), bound together by a disulfide bridge. Unlike DT or TT, CRM197 does not need detoxification with formaldehyde; homogeneous formulations of purified antigens can be instantly acquired [58-61].

Tetanus Toxoid

It is frequently used as a carrier protein for TCV, adding to being a component of the DPT vaccine. Clostridium tetani cultures produce tetanus toxin, detoxified by formaldehyde, and produce TT. Studies reported that Vi-TT is a highly immunogenic (with a seroconversion rate of 100% when compared with Vi-PS [30]) vaccine that considerably reduces typhoid fever cases when evaluated using a stringent controlled model of typhoid infection. Typhoid fever and associated health inequalities can be reduced with Vi-TT [26]. See Table 2 for a comparative overview between TT and CRM197.

**Table 2:** TT versus CRM197 TT, tetanus toxoid, CRM197, cross-reacting material

TT	CRM197
Conjugates based on TT and DT proved to be protective against a lethal challenge by tetanus toxin or diphtheria toxin after only one injection, even when the vaccine has not been adjuvanted [62].	Analogous quadrivalent CRM197-based MenACWY vaccine was not protective after one injection or two immunizations. Failure of protection was also revealed by an adjuvanted CRM197-based monovalent conjugate [62].
According to Broker et al., when a group of immunologically naïve mice was immunized with conjugate vaccine MenC_TT/PRP_TT, containing a total amount of 10.5 mg of TT per dose, all mice were protected after one injection against a lethal challenge by TT [62].	According to Broker et al., when CRM197 is used as a carrier protein, it can defend against a lethal challenge by diphtheria toxin. The MenACWY_CRM vaccine was assessed, and animals received one dose of vaccine with 8 mg of CRM197. None of the guinea pigs survived the challenge and raised the CRM197 concentration per dose to 44 mg (human dose) and administered 2 doses with a time interval of 14 days, which did not confer protection [62].

Serological responses to TCV

A sero-efficacy study can demonstrate the efficacy of new conjugate vaccines without expensive surveillance programs for typhoid fever and without enrolling thousands of participants [63].

Over the last century, serologic tests for typhoid have existed, but sensitive and specific antigens have only recently been identified. Combining these antigens with new methodological development does not appear to modify the subject's cross-sectional serosurveys. As a result of these new tools, a wide range of applications for enteric fever sero-epidemiology are now possible, including high-resolution surveillance data generation, vaccine impact monitoring, and integration with other sero-surveillance programs [64]. Recent studies have evidence of the serological response to TCV.

Khanam et al. [65] in a recent randomized Vi-TT trial using the JE vaccine as the control evaluated immune response in 1,500 children randomly selected on a 2:1 basis (Vi-TT vs JE vaccine). The study reported that a robust, persistent antibody response was induced after a single dose of Vi-TT, even after two years of vaccination. Jin et al. [66], in 2020, conducted a phase IIb, a blinded, randomized controlled trial in adults. Participants were vaccinated with Vi-TT or Vi-PS vaccines and were subsequently challenged with *S. typhi*. The results reported that significantly higher Vi-specific humoral responses were induced 28 days after vaccination compared with baseline for both Vi-TT and Vi-PS vaccines. Vi-TT induces significantly higher fold increases in Vi-specific IgG responses than Vi-PS, and Vi IgA and IgG responses predict protection from typhoid fever. Lee et al. [67] enrolled 48 serum samples from adults and children; participants were randomized to receive Vi-DT or Typhim Vi (plain Vi). Study results revealed a good correlation between the two assays when the anti-Vi IgG titer was assessed using VaccZyme ELISA based on the Vi-IgGR1 (P < 0.001) or VaccZyme ELISA calibrator (P < 0.001). Comparable estimations of anti-Vi IgG geometric mean titer (GMT) were observed after vaccination with the Vi-DT (1,626 EU/mL) and Vi-TT vaccines (1,293 EU/mL). Table 3 illustrates the comparison of different TCVs available based on immunogenicity.

**Table 3:** Comparison of different TCVs available based on immunogenicity, attributes, and AEFI AEFI, adverse events following immunization; CRM197, cross-reacting material (nontoxic mutant of diphtheria toxin); EPI, expanded program on immunization; IgG, immunoglobulin G; TCV, typhoid conjugate vaccine; TT, tetanus toxoid; Vi, Vi polysaccharide

Types of TCVs Available	Carrier Protein	Conjugated Method	Immunogenicity	Attributes	AEFI
Prototype conjugated protein-Vi-rEPA [27,68]	The non-toxic recombinant protein (Pseudomonas aeruginosa exotoxin A)	Hetero-bifunctional cross-linking reagent (N-succinimidyl-3-(2-pyridyldithio)-propionate or adipic acid dihydrazide)	Vi-rEPA elicits a T-cell-dependent immune response, leading to affinity maturation, subclass switching, and induction of memory, leading to a long-lasting immune response against the Vi polysaccharide of *S. typhi*.	Booster response: children aged 2 to 4 years showed a booster response, resulting in higher IgG anti-Vi levels than Vi polysaccharide alone in children aged 5 to 14. Efficacy: in a double-blind, placebo-controlled, randomized efficacy study in Vietnam, the vaccine showed an overall efficacy of 89% in children aged 2 to 5 years. Safety in infants: the vaccine is safe and compatible with EPI vaccines in infants. Protective IgG anti-Vi levels were observed in 95% of infants at 13 months after the fourth injection.	No local reactions or fever (>38.5^°^C)
Typbar-TCV® (Bharat Biotech) [27]	TT	Conjugation of Vi polysaccharide to TT carrier protein	Highly immunogenic in all age groups. Results in higher IgG anti-Vi levels and avidity compared to Vi polysaccharide alone. Induces multiple IgG subclasses and strong booster responses in all age groups.	Licensed for use from age ≥six months to 45 years. Induces booster response in children aged 2 to 4 years. Exhibits high efficacy against typhoid fever in adults (87.1%). Satisfactory antibody response and memory with increased protection. WHO prequalified for use (January 2018).	No vaccine-attributable serious adverse events were observed. Successful co-administration with measles vaccine
PedaTyph (BioMed) [27,31]	TT	Conjugation of Vi polysaccharide to TT carrier protein	Seroconversion rate (≥4-fold increase over preimmunization titer) of 83% in children aged <2 years at 8 weeks post-vaccination. Substantial seroconversion rates after six weeks (100%) and 12 months (83%) in a subgroup evaluation.	Licensed as the first TCV in India. Induces strong and long-lasting immune response with higher IgG titers at 30 months post-vaccination. Efficacy of 100% in the first year of follow-up. Recommended for children aged >3 months as a single dose of 0.5 mL followed by boosters at age 2.5 to 3 years.	Minimal adverse events post-vaccination
Zyvac TCV (Zydus Lifesciences Ltd) [8,27]	TT	Conjugation of Vi polysaccharide to TT carrier protein	Demonstrated immune non-inferiority with Typbar-TCV® in phase 2 and 3 clinical trials (238 participants in all age groups)	Licensed as a single dose of 25 µg from age six months onward in India	No specific AEFI reported
Vi-CRM197 (GVGH) [69]	CRM197 (non-toxic mutant of diphtheria toxin)	Conjugation of Vi polysaccharide to CRM197 carrier protein	Phase 1 and 2 trials demonstrated safety and immunogenicity in European adults, comparable to Vi-PS. Responses were short-lived in the phase 2 trial with 320 participants in endemic populations.	Considered safe and immunogenic in endemic populations of all ages in India, Pakistan, and the Philippines. Can be given concomitantly with the measles vaccine at age nine months, potentially suitable for inclusion in EPI schedules. Technology transferred to Biological E. Ltd and is in full clinical development.	No specific AEFI reported

Need of number of doses and booster doses of TCV

In the case of TCV, the number of doses needed for a primary series is unknown. The protocols of some early TCV trials used more than one primary dose [70,71]. Canh et al. reported that both one and two-dose recipients of the Vi-rEPA vaccine showed comparable point estimates of efficacy (87.7 and 89%, respectively) after 46 months of vaccination [71]. Bhutta et al., in the multicentric trial in Pakistan, India, and the Philippines, reported that the second dose of Vi-CRM in the primary series did not increase GMTs in children and older infants [72]. In a subset analysis conducted by Chinnasami et al., it was reported that single-dose PedaTyphTM was not adequate for seroconversion, and children below the age of two years were found to elicit better response than the older ones [73]. Thus, to these findings, several questions still need to be answered regarding the exact doses needed for a primary series of Vi-TCV.

According to the available study results on licensed TCVs in India, a single dose should be able to provide the required protection for a time period of at least 2.5 to 5 years. As per available data, one dose appears sufficient to induce an adequate immune response, and primary series with closely spaced doses will not confer higher immunity [27]. Nevertheless, some studies have observed some waning of antibody titers after 6 to 12 months of immunization [74]. A booster dose may be needed in children (<2 years). Though for endemic countries, a single dose of TCV, at any time between 6 and 23 months of age, was recommended by the WHO-SAGE working group on typhoid vaccines [24].

TCV and public health in India

Typhoid remains a significant public health concern in India, leading to a considerable burden of morbidity and mortality. India has made significant strides in introducing TCVs into its immunization program to combat this issue. Currently, there are three Indian-licensed, commercially available TCVs: Typbar-TCV (Bharat Biotech), ZyVac TCV (Zydus Lifesciences Ltd), and the recently licensed Vi-CRM197 (Biological E. Ltd, Hyderabad, India) [8].

 In India, the Navi Mumbai Municipal Corporation (NMMC) has introduced TCV into its immunization program, targeting children aged nine months to 14 years in 11 of 22 areas (Phase 1 campaign) in 2018. This first public sector TCV campaign was successfully implemented by NMMC, with high administrative coverage in slums and low-income areas [75].

In India, the Ty21a oral typhoid vaccine is used to combat typhoid fever, a live attenuated vaccine administered as capsules or enteric-coated tablets. It effectively prevents the disease, particularly in areas with limited healthcare access, and its ease of administration allows for mass campaigns and routine immunization. Multiple doses establish lasting immunity by stimulating the body's response to weakened *S. typhi*. The vaccine is generally safe, with mild, transient side effects. Combined with TCVs, India aims to reduce typhoid burden through proper vaccination, sanitation, and hygiene practices emphasizing the importance of public awareness and surveillance for successful prevention strategies [76,77].

TCV and global demand

In the current global typhoid vaccine market, 96% of the demand for TCV comes from childhood vaccination programs [78]. The TCV market is currently characterized by uncertainty surrounding the uptake and introduction of the vaccine. Several factors contribute to this uncertainty, including insufficient epidemiological data to support decision-making for TCV introduction and immunization strategies. Three estimates of TCV demand evolution have been developed to capture 2021-2030 market variability. The higher estimate assumes widespread adoption with aggressive national strategies, while the lower estimate anticipates limited introduction due to less interest [78].

Global mid-term demand for TCV is estimated to range from 43 million to 163 million doses per year (moderate estimate of 111 million doses per year). It is expected to decrease to 22-96 million doses per year (a moderate estimate of 71 million doses) once the one-time campaigns have been conducted. India's demand for TCVs is estimated at 24 million doses in 2030, according to the moderate estimate [78].

TCV induction in endemic areas

WHO SAGE proposed TCV induction, and Gavi funded $85 million. In 2019, Zimbabwe launched a TCV campaign that reduced typhoid cases. Pakistan introduced TCV in its routine immunization schedule and vaccinated 23 million children. Liberia introduced TCV in a non-epidemic setting and integrated it into the regular immunization program. Zimbabwe started its massive national TCV campaign in May 2021, aiming to vaccinate 6.2 million children. Currently, many nations in Africa and Asia are at various stages of the TCV application and introduction processes.

Adverse events following immunization with the current typhoid conjugate vaccines

Typbar-TCV (Bharat Biotech) and Zyvac TCV (Zydus Lifesciences Ltd) are TCVs that have been extensively studied for their safety and immunogenicity. Both vaccines have demonstrated a favorable safety profile and are well-tolerated. Mild and transient local reactions, such as pain, redness, and swelling at the injection site, have been reported for both vaccines. Systemic responses such as fever, headache, and fatigue were also observed but were generally self-limiting and of short duration [8,25,31].

The vast majority of adverse events following immunization associated with Typbar-TCV and Vi-TT were in mild-to-moderate intensities, which resolved spontaneously without the need for any specific rescue medications. Serious adverse events were rare for both vaccines and not causally associated with the vaccination [25,31].

Typbar-TCV received WHO prequalification in January 2018, affirming its safety and efficacy. Conversely, Vi-TT has undergone clinical trials for immune non-inferiority with Typbar-TCV and has also been licensed for use in India [8,25].

Given the similar carrier protein (TT) used in both vaccines, Vi-TT's safety profile is expected to be comparable to Typbar-TCV, supporting the notion of non-inferiority between the two vaccines regarding safety and immunogenicity [25,31]. All the pros and cons of TCV available in our current knowledge pool are summarized in Table 4.

**Table 4:** Pros and cons of TCV AMR, antimicrobial resistance; TCV, typhoid conjugate vaccine

Pros	Cons
Efficacy: TCVs have higher efficacy, have a longer duration of protection, and are safe and immunogenic in children as young as 6 months old, making TCVs compatible with existing infant immunization programs [79]. Cost-effectiveness: studies reported that TCV is a cost-effective vaccine. One study reported routine infant vaccination at $1/dose was cost-saving in Delhi and Dong Thap, "very cost-effective" in Kolkata and Nairobi, and "cost-effective" in Lwak according to WHO thresholds [80]. Due to an increase in AMR, TCVs may help reduce mortality and morbidity [30].	Inability to provide protection against S. Paratyphi A and also against non-typhoidal Salmonella serotypes due to the formulation, which is based on Vi-antigen; thus, TCVs are ineffective against S. Typhi strains, which are Vi-negative and exist naturally [81]. Although serious adverse events are rare, studies reported that adverse events following immunization with TCV are common [41,70].

## Conclusions

In addition to other preventive measures, a comprehensive vaccination strategy is needed to reduce the burden of typhoid in endemic regions. This burden remains unaddressed by the Vi-PS vaccines; thus, new-generation TCVs are the most effective way to overcome the limitations. TCVs are safe for children, and they do not interfere with routine vaccination schedule currently operational in the typhoid endemic countries. Above all, TCVs were found to elicit long-term protection and minimize the need for re-vaccination. Thus, the new TCVs are being implemented and introduced in several African and Asian nations. The current generation of TCVs is usually considered safe, effective, and immunogenic, and offers protection for extended periods. However, even after having so many advantages, TCVs require extensive post-marketing surveillance studies, including a larger sample size.
